# Development and evaluation of machine learning models for predicting large-for-gestational-age newborns in women exposed to radiation prior to pregnancy

**DOI:** 10.1186/s12911-024-02556-6

**Published:** 2024-06-20

**Authors:** Xi Bai, Zhibo Zhou, Zeyan Zheng, Yansheng Li, Kejia Liu, Yuanjun Zheng, Hongbo Yang, Huijuan Zhu, Shi Chen, Hui Pan

**Affiliations:** 1 Key Laboratory of Endocrine Glucose & Lipids Metabolism and Brain Aging, Department of Endocrinology, Ministry of Education, Shandong Provincial Hospital Affiliated to Shandong First Medical University, Jinan, Shandong China; 2 Key Laboratory of Endocrinology of National Health Commission, Department of Endocrinology, State Key Laboratory of Complex Severe and Rare Diseases, Peking Union Medical College Hospital, Chinese Academy of Medical Science and Peking Union Medical College, Beijing, 100730 China; 3 DHC Mediway Technology CO., Ltd, Beijing, China

**Keywords:** Large-for-gestational-age, Exposed to radiation, Machine learning, Prediction model

## Abstract

**Introduction:**

The correlation between radiation exposure before pregnancy and abnormal birth weight has been previously proven. However, for large-for-gestational-age (LGA) babies in women exposed to radiation before becoming pregnant, there is no prediction model yet.

**Material and methods:**

The data were collected from the National Free Preconception Health Examination Project in China. A sum of 455 neonates (42 SGA births and 423 non-LGA births) were included. A training set (*n* = 319) and a test set (*n* = 136) were created from the dataset at random. To develop prediction models for LGA neonates, conventional logistic regression (LR) method and six machine learning methods were used in this study. Recursive feature elimination approach was performed by choosing 10 features which made a big contribution to the prediction models. And the Shapley Additive Explanation model was applied to interpret the most important characteristics that affected forecast outputs.

**Results:**

The random forest (RF) model had the highest average area under the receiver-operating-characteristic curve (AUC) for predicting LGA in the test set (0.843, 95% confidence interval [CI]: 0.714–0.974). Except for the logistic regression model (AUC: 0.603, 95%CI: 0.440–0.767), other models’ AUCs displayed well. Thereinto, the RF algorithm’s final prediction model using 10 characteristics achieved an average AUC of 0.821 (95% CI: 0.693–0.949).

**Conclusion:**

The prediction model based on machine learning might be a promising tool for the prenatal prediction of LGA births in women with radiation exposure before pregnancy.

**Supplementary Information:**

The online version contains supplementary material available at 10.1186/s12911-024-02556-6.

## Introduction

Babies born large for gestational age (LGA) are defined as birth weight > 90th percentile according to gestational age and sex [1]. Previous studies found that LGA births were related to a higher risk of adverse pregnancy outcomes, including shoulder dystocia, postpartum hemorrhage, cesarean section, neonatal hypoglycemia and longer hospital stay [2, 3]. It is clear that LGA births improved the chance of stillbirth and perinatal death [4–6]. As the birth weight percentile rises, the above risks increase. Additionally, being LGA newborns is also associated with increased long-term risk of obesity, type 2 diabetes, childhood primary brain tumors and multiple adult cancers in their lives [7–12]. Recently, LGA births are also reported as a well-performed classifier for the risk of adverse perinatal outcomes [13]. If the LGA births can be recognized before delivery, early intervention, closer monitoring and targeted perinatal medical care can be performed to decrease adverse composite outcomes. Thus, a prenatal prediction of LGA births is of vital importance, especially in the susceptible populations of LGA pregnancies.

Numerous factors influence LGA births since birth weight is a composite result affected by different genetic and environmental factors. The maternal risk factors for abnormal birth weight include obesity, gestational diabetes mellitus, older age and so on [14–16]. Besides, radiation exposure before pregnancy may induce significant damage in ovary and uterus [17–19]. Many studies have proved the correlation between radiation before pregnancy and abnormal birth weight [20, 21]. However, there is still no model for LGA birth prediction in women who were exposed to radiation before becoming pregnant.

Prediction models on the basis of conventional statistical methods are not good at dealing with multiple variables in large datasets, for which ignore the potential relationships among multiple variables [22]. Machine learning (ML) had been widely used in prediction models in recent years, for its advantages of modelling complex interactions from multiple variables in large datasets and requiring no model specification [23, 24]. As for LGA births prediction, previous studies tried to develop prediction models using ML based on maternal factors in the general population, but most of them perform poorly [25–28]. In recent years, many environmental factors and paternal factors were proven as risk factors for LGA births, including second-hand smoking exposure, pregnancy PM2.5 exposure, advanced paternal age, higher paternal height and so on [29–32], but they had not been included into the existing prediction models.

This study aims to develop and evaluate prediction models for LGA births in women with radiation exposure before pregnancy by using different ML algorithms. This study was the first study to develop prediction models in women with radiation exposure, being based on the National Free Preconception Health Examination Project (NFPHEP) in China, a nationwide prospective cohort including maternal, paternal and environmental factors. Moreover, the paternal and environmental factors were innovatively integrated into the LGA prediction models as predictive factors for the first time.

## Materials and methods

### Data source

This study was performed based on the data from the NFPHEP, a three-year nationwide project including more than 240,000 newborns from Jan. 2010 to Dec. 2012, which was initiated by the National Health Commission of the People’s Republic of China and carried out in over 220 counties across 31 provinces/municipalities in China [33]. The study design and conducting of the NFPHEP had been previously reported in details [33–35]. In general, the preconception health condition and risk factors for adverse pregnancy outputs were investigated in the NFPHEP, to increase the pregnant women’s overall health and neonates. All data in the NFPHEP had been uploaded to a nationwide electronic data collecting system, and the quality control was performed by The National Quality Inspection Center for Family Planning Techniques. The NFPHEP protocol (protocol code 2,017,101,702) was authorized by the Institutional Review Committee of the National Research Institute for Family Planning in Beijing, China. All the participants and their legal guardians signed informed consent form.

### Study participants and features

All singleton live neonates with gestational age of over 24 weeks and complete birth records were selected from the NFPHEP, and then 985 cases whose mothers were radioactively contaminated in working or living environment before pregnancy were included in this study. After deleting the records with omitted values or extreme values of demographic features, the last analysis comprised 455 cases, including 42 LGA births (9.23%) and 413 non-LGA births (91.77%). Experiments were performed for free during pre-pregnancy, pregnancy and postpartum follow-up. A total of 153 features about the maternal/paternal social demographic characteristics, lifestyle, social economic status, family history, pre-existing medical problems, physical and laboratory examinations, and neonatal birth information were obtained through face-to-face questionnaires and experiments conducted by specific staffs who received standardized training. In this study, LGA was defined as neonates having a birth weight over the 90th percentile for their gender and gestational age [36].

### Study design

**Fig. 1:**
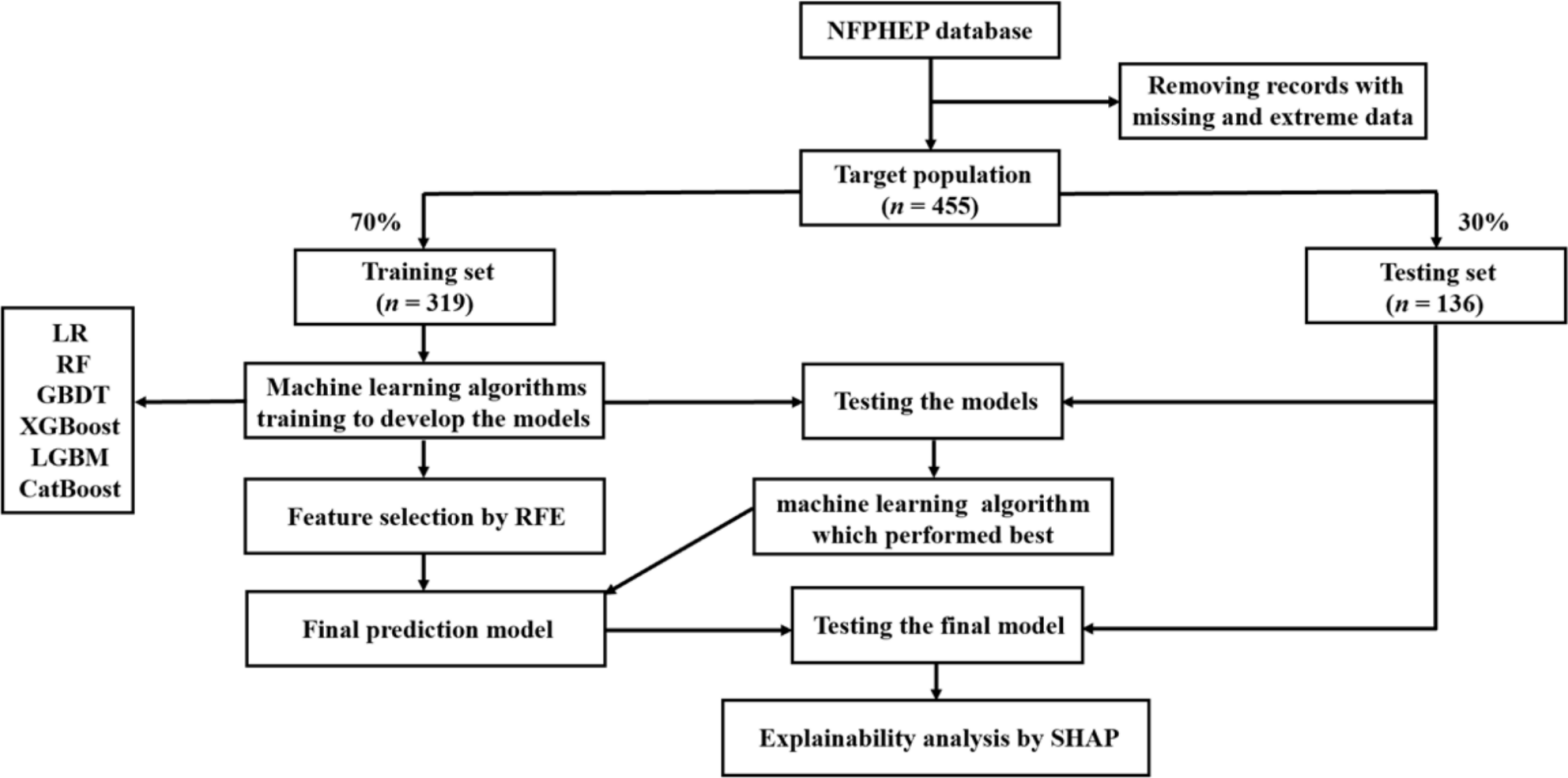
The flow chart of the methods in this study, including data extraction, training, and testing. A total of 455 participants were included in this study, which were divided into training dataset and testing dataset. *Abbreviations* NFPHEP = National Free Preconception Health Examination Project, LR = Logistic Regression, RF = Random Forest, GBDT = Gradient Boosting Decision Tree, XGBoost = Extreme Gradient Boosting, LGBM = Light Gradient Boosting Machine, CatBoost = Category Boosting, RFE = Recursive Feature Elimination, SHAP = Shapley Additive Explanation

The study design and data processing flow were shown in the flow chart as Fig. 1. All analyses in this study were conducted in Python (version 3.8.5). The dataset (*n* = 455) was split into a training set (70%, *n* = 319) and a test set (30%, *n* = 136) for the development and evaluation of the ML prediction models for LGA. ML prediction models were developed and evaluated as described in our previous study [37]. In brief, 153 related characteristics (shown in Table S1) were contained as candidate predictor variables in six algorithms, including logistic regression (LR), random forest (RF), gradient boosting decision tree (GBDT), extreme gradient boosting (XGBoost), light gradient boosting machine (LGBM), and category boosting (CatBoost). The performances of these models were evaluated by area under the receiver operating characteristic (ROC) curve (AUC, main evaluating index), sensitivity, specificity, positive predictive value (PPV), and negative predictive value (NPV). The RF approach was selected to develop the final model because of its highest average AUC value in test set among all algorithms (shown in Results). To reduce the computational cost in developing the final model, the recursive feature elimination (RFE) method was performed to choose 10 characteristics which made an important contribution to the LGA prediction output from the 153 candidate features, using a RF classifier as the estimator. For the ML algorithm (RF) with the highest average AUC, the hyperparameters were set as n_estimators = 30, max_depth = 4, and min_samples_split = 0.15. The effectiveness of the RFE had been reported in many medical studies [38–41]. Thus, the final model was developed, including the above 10 features using the RF algorithm. In addition, to explain the final model, the Shapley Additive Explanation (SHAP) approach was used to use the post hoc explain the ability of the final model, to interpret the impact of all contained characteristics. SHAP is a useful game theory method to assess the significance of the specific input attributes to the prediction outcome [42].

### ML algorithms

A total of six algorithms were employed to improve the prediction models which had been described in our previous study, including LR, RF, GBDT, XGBoost, LGBM, and CatBoost [37, 43]. Overall, traditional LR approach and other five methods are the most prevalent and state-of-the-art supervised machine learning approaches for categorization problems. In brief, the LR algorithm is commonly used in medical research, which can evaluate the probability of a binary dependent variable [44]. The RF algorithm is an ensemble classification process, which can combine multiple decision trees by majority voting [45, 46]. The GBDT algorithm on the basis of the ensembles of decision trees is known due to its reliable, effectiveness, and comprehensibility. For each step, there is a novel determination being trained to match the residual between the ground truth and the current prediction [47]. The XGBoost algorithm can use the second-order gradient to improve the approximation greedy search, the parallel learning, and the hyperparameters which can reduce the problems of overestimation and underestimation [48]. The LGBM algorithm can greatly increases the training efficiency by using a histogram to aggregate gradient information [49]. And the CatBoost algorithm uses a novel approach to cope with categorical features that reduce the issue of gradient bias as well as prediction shift [50].

### Statistical analysis

Categorical variables in this study were expressed by numbers (%) using either the Chi-square test or Fisher’s exact test. The Wilcoxon Mann-Whitney U test was utilized to compare data that are constant but do not follow a normal distribution. Continuous variables that did fit a normal distribution were reported as median (interquartile range [IQR]) and compared based on the two-tailed Student’s t-test. Additionally, each model’s AUC, sensitivity, specificity, PPV, and NPV were assessed. The AUC in training and test sets was primarily used to assess the prediction abilities of the ML models. Statistical significance was defined as a two-sided *P*-value of 0.05. Python was used to perform all statistical analyses.

## Results

### Demographic features

The NFPHEP database recorded 455 neonates whose mothers had radiation exposure from working and living surroundings ahead of pregnancy from Jan. 2010 to Dec. 2012. They were divided into two groups, including 42 LGA births (9.23%) and 413 non-LGA birth (91.77%). The demographic characteristics were shown in Table 1. Overall, the neonates possessed a median gestational age of 40.0 weeks (IQR: 39.0,40.0) and a birth weight of 3.30 kg (IQR: 3.00,3.55). Expectedly, LGA newborns had a significantly higher birth weight than non-LGA neonates (4.05 kg vs. 3.25 kg, *P* < 0.001). There were no differences in maternal or paternal age, height, body mass index (BMI) and diastolic blood pressure (DBP) ahead of pregnancy between non-LGA group and LGA group. While those mothers of LGA neonates had a significantly lower frequency of taking folacin regularly (64.29% vs. 79.66%, *P* = 0.02), compared to those of non-LGA. Besides, those fathers of LGA neonates had higher systolic blood pressure (120mmHg vs. 115mmHg, *P* = 0.035) and a significantly increased frequency of suffering from economic pressure (45.24% vs. 36.56%, *P* = 0.016) or life/work pressure (57.14% vs. 35.35%, *P* = 0.009) ahead of pregnancy, compared to those of non-LGA. In addition, the results on comparing 153 variables for predictors were detailed displayed in Table S1 from Supplementary.

**Table 1:** Part of demographic characteristics of the subjects included in analysis

Characteristics	Overall (*n* = 455)	Non-LGA(*n* = 413)	LGA(*n* = 42)	*P* value
Male gender	246(54.07%)	227(54.96%)	19(45.24%)	0.297
Gestational at birth, week	40.0[39.0, 40.0]	40.0[39.0, 40.0]	39.0[39.0, 40.0]	0.065
Birth weight, kg	3.30[3.00, 3.55]	3.25[3.00, 3.50]	4.05[3.95, 4.33]	< 0.001
Maternal age, year	24.0[23.0, 27.0]	24.0[23.0, 27.0]	24.5[23.0, 26.0]	0.273
Maternal height, cm				
< 150 cm	3(0.66%)	3(0.73%)	0(0.00%)	0.948
150–159 cm	221(48.57%)	200(48.43%)	21(50.00%)	
160–169 cm	218(47.91%)	198(47.94%)	20(47.62%)	
≥ 170 cm	13(2.86%)	12(2.91%)	1(2.38%)	
Maternal BMI, kg/m^2^	20.2[18.78, 22.05]	20.2[18.82, 22.03]	20.115[18.38, 22.56]	0.393
Maternal SBP, mmHg	108.167 ± 10.655	108.034 ± 10.519	109.476 ± 11.97	0.404
Maternal DBP, mmHg	70.0[68.0, 75.0]	70.0[68.0, 75.0]	70.0[62.25, 79.0]	0.424
Maternal life/work pressure				
None	267(58.68%)	247(59.81%)	20(47.62%)	0.228
Mild	172(37.8%)	151(36.56%)	21(50.00%)	
Severe	16(3.52%)	15(3.63%)	1(2.38%)	
Maternal economic pressure				
None	315(69.23%)	289(69.98%)	26(61.90%)	0.402
Mild	131(28.79%)	117(28.32%)	14(33.33%)	
Severe	9(1.98%)	7(1.69%)	2(4.76%)	
Maternal taking folacin regularly				
Regularly	356(78.24%)	329(79.66%)	27(64.29%)	0.020
Irregularly	32(7.03%)	25(6.05%)	7(16.67%)	
Not taking	67(14.73%)	59(14.29%)	8(19.05%)	
Paternal age, year	26.0[24.0, 29.0]	26.0[24.0, 29.0]	26.0[24.0, 27.75]	0.088
Paternal height, cm				
<160 cm	3(0.66%)	3(0.73%)	0(0)	0.954
160–169 cm	135(29.67%)	122(29.54%)	13(30.95%)	
170–179 cm	284(62.42%)	258(62.47%)	26(61.9%)	
≥ 180 cm	33(7.25%)	30(7.26%)	3(7.14%)	
Paternal BMI, kg/m^2^	22.04[20.28,24.49]	22.04[20.28,24.49]	22.12[19.92,24.48]	0.326
Paternal SBP, mmHg	117.0[110.0, 120.0]	115.0[110.0, 120.0]	120.0[110.0, 125.0]	0.035
Paternal DBP, mmHg	75.0[70.0, 80.0]	75.0[70.0, 80.0]	76.0[70.0, 80.0]	0.175
Paternal life/work pressure				
None	285(62.64%)	267(64.65%)	18(42.86%)	0.009
Mild	152(33.41%)	132(31.96%)	20(47.62%)	
Severe	18(3.96%)	14(3.39%)	4(9.52%)	
Paternal economic pressure				
None	285(62.64%)	262(63.44%)	23(54.76%)	0.016
Mild	154(33.85%)	139(33.66%)	15(35.71%)	
Severe	16(3.52%)	12(2.90%)	4(9.52%)	

*Abbreviations* LGA = Large for Gestational Age, BMI = Body Mass Index, SBP = Systolic Blood Pressure, DBP = Diastolic Blood Pressure. The above data were presented as number (%), median [interquartile range] or mean ± standard deviation. And Continuous variables are compared by the Student’s t-test or Wilcoxon Mann–Whitney U test. Categorical variables are compared by Chi-square or Fisher’s exact test

### ML algorithms’ performance comparison

**Fig. 2:**
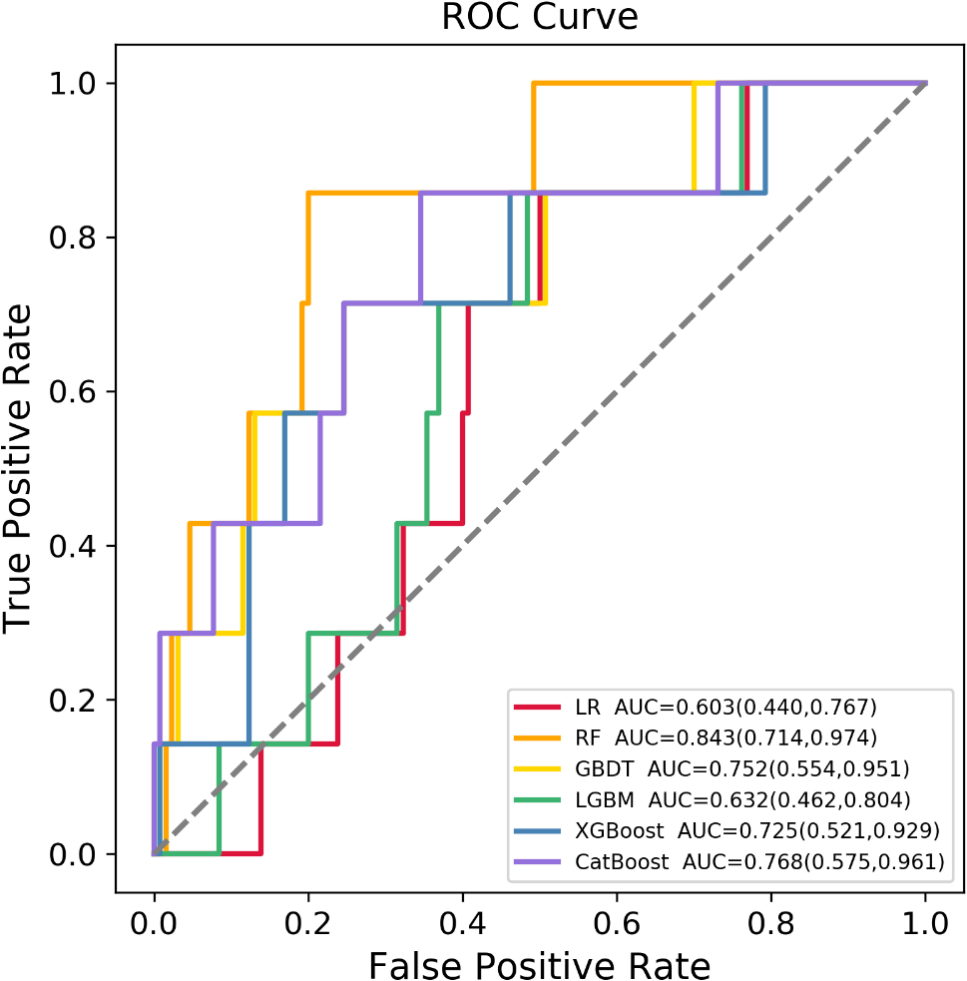
ROC curves of the above 6 machine learning models for predicting LGA in the test set. The RF model achieved the top average AUC value (AUC = 0.843, 95%CI: 0.714–0.974) among above models. *Abbreviations* ROC = Receiver Operating Characteristic, LGA = Large for Gestational Age, Area Under, LR = Logistic Regression, AUC = The Receiver Operating characteristic Curve, RF = Random Forest, GBDT = Gradient Boosting Decision Tree, LGBM = Light Gradient Boosting Machine, XGBoost = Extreme Gradient Boosting, CatBoost = Category Boosting

The training set (*n* = 319) was utilized for LGA birth based on LR, RF, GBDT, XGBoost, LGBM, and CatBoost. The test set (*n* = 136) was also utilized to assess the effectiveness of their LGA prediction models. Figure 2 illustrates the comparison on the ROC curve for LGA prediction in the 6 improved models using the test set. Therefore, the RF model had the highest average AUC value (0.843, 95% confidence interval [CI]: 0.714–0.974) to predict LGA in the test set. And other models also showed a good average AUC in the test set: GBDT (AUC: 0.752, 95% CI:0.554–0.951), XGBoost (AUC:0.725, 95%CI: 0.521–0.929), CatBoost (AUC: 0.768, 95%CI:0.575–0.961), except for LR (AUC:0.603, 95%CI:0.440–0.767) and LGBM (AUC:0.632, 95%CI:0.462–0.804). Besides, sensitivity, specificity, PPV, and NPV in the above models ranged from 0.714 to 1.000, 0.500 to 0.800, 0.085 to 0.188, and 0.980 to 0.990, respectively. And more information was listed in Table 2, which included AUC values from both training set and the test set, sensitivity, specificity, PPV, and NPV in each model.

**Table 2:** Performance of models by different algorithms in predicting large for gestational age (LGA) neonates

Model	AUC training	AUC testing	Sensitivity	Specificity	PPV	NPV
LR	0.965	0.603	0.857	0.500	0.085	0.985
RF	0.950	0.843	0.857	0.800	0.188	0.990
GBDT	0.980	0.752	0.714	0.754	0.135	0.980
XGBoost	0.999	0.725	0.714	0.754	0.135	0.980
LGBM	0.937	0.632	0.857	0.515	0.087	0.985
CatBoost	0.979	0.768	0.857	0.654	0.118	0.988

*Abbreviations* AUC = Area Under the Receiver-Operating-Characteristic Curve, PPV = Positive Predictive Value, NPV = Negative Predictive Value, LR = Logistic Regression, RF = Random Forest, GBDT = Gradient Boosting Decision Tree, XGBoost = Extreme Gradient Boosting, LGBM = Light Gradient Boosting Machine, CatBoost = Category Boosting

### Characteristics choosing and model prediction

**Fig. 3:**
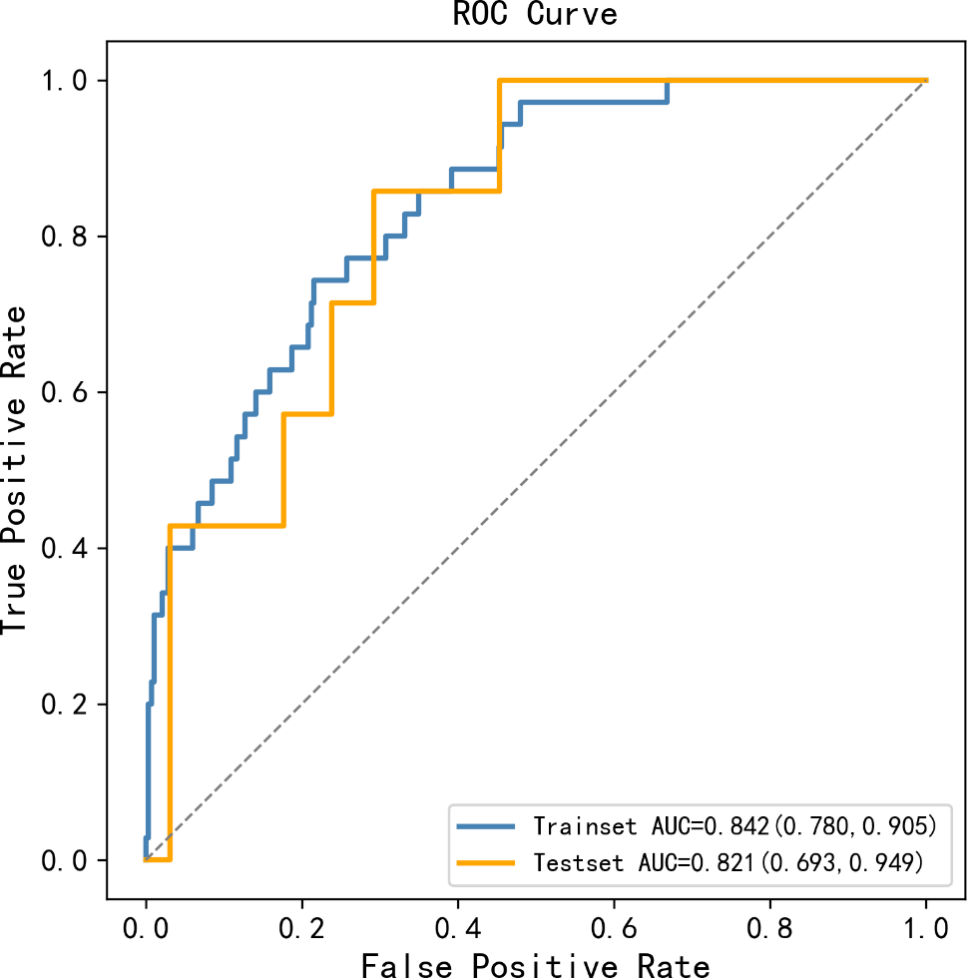
ROC curves of the final machine learning model for predicting LGA in training set (AUC = 0.842, 95%CI:0.780–0.905), and test set (AUC = 0.821, 95%CI:0.693–0.949). The final predicting model was based on the random forest algorithm, and included top 10 contributed features chosen by RFE method. *Abbreviations* ROC = Receiver Operating Characteristic, LGA = Large for Gestational Age, AUC = The Receiver Operating Characteristic Curve, RFE = Recursive Feature Elimination

To lower the computational expense in developing models, the RFE method was performed to select 10 features which considerably influenced the outcome of the prediction using the 153 candidate features. These features include paternal alanine aminotransferase (ALT) ahead of pregnancy, maternal creatinine (Cr) ahead of pregnancy, paternal work/life pressure ahead of pregnancy, paternal heartrate ahead of pregnancy, paternal Cr ahead of pregnancy, maternal meat/eggs diet ahead of pregnancy, maternal hepatitis B virus e antigen (HBeAg) ahead of pregnancy, maternal ALT ahead of pregnancy, maternal DBP ahead of pregnancy, physical examination for maternal thyroid ahead of pregnancy. Thus, these 10 features were utilized to develop the final prediction model based on the RF algorithm which reached the top average AUC value in test set. And the result of final model’s ROC curve in the training and test set for LGA prediction were displayed in Fig. 3. Specifically, AUC values in both sets were 0.842 (95%CI:0.780–0.905) and 0.821(95%CI: 0.693–0.949), and the sensitivity, specificity, PPV, and NPV of the final model were 0.857, 0.708, 0.136 and 0.989, separately.

### Assessment of variable importance

**Fig. 4:**
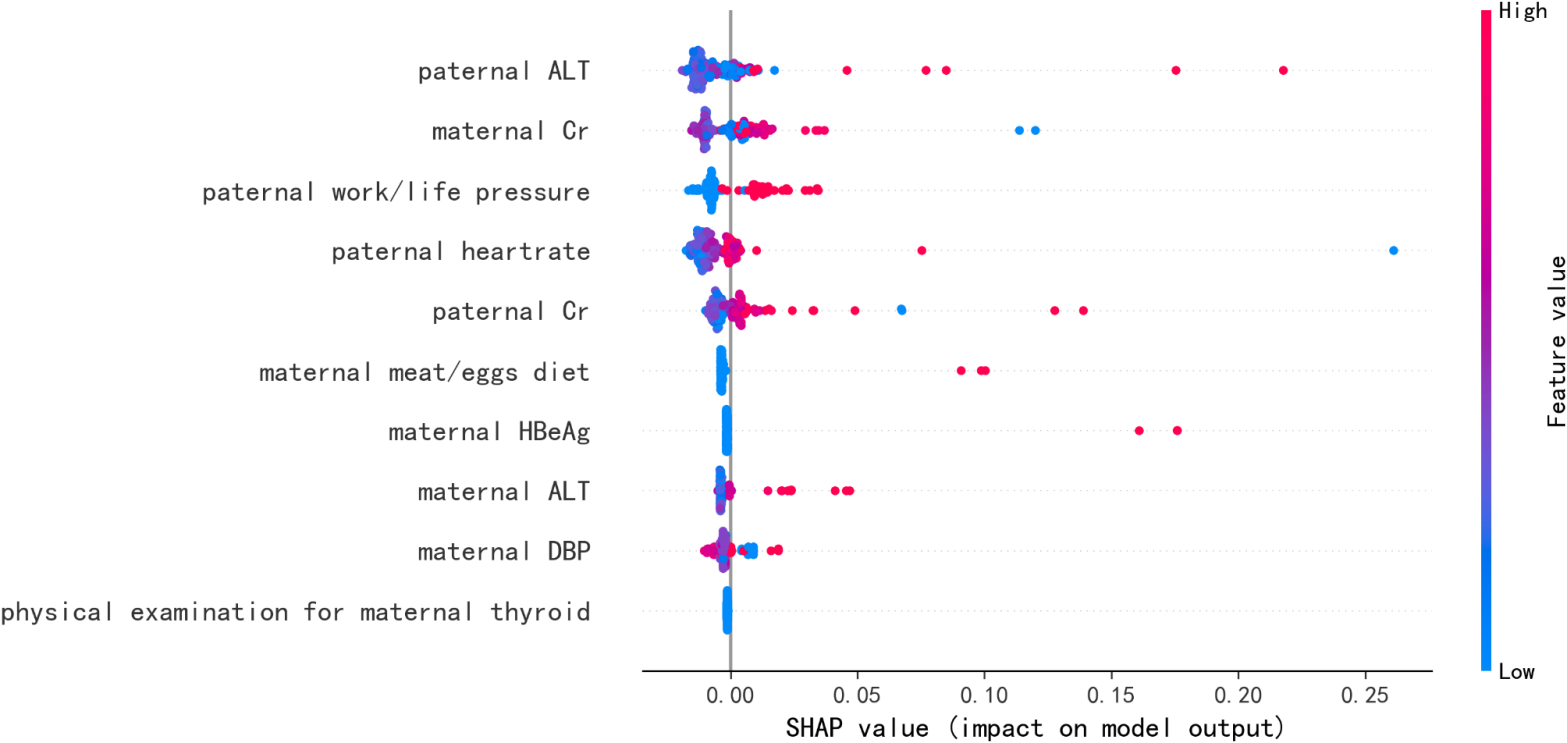
The SHAP values for most important predictors of LGA in the final model. The Y-axis showed the importance of each feature from top to bottom, and the X-axis showed the mean SHAP values. Each line represented a feature, and each dot in the diagram represented a sample in the cohort. Hot color (red) indicates a high value for this feature, while cold color (blue) indicates a low value for this feature. *Abbreviations* SHAP = Shapley Additive Explanation, LGA = Large for Gestational Age, ALT = Alanine Aminotransferase, Cr = Creatinine, HBeAg = Hepatitis B Virus e Antigen, DBP = Diastolic Blood Pressure

To identify the features which greatly influence on the final prediction model, the SHAP summary diagram of the final model was drawn and shown in Fig. 4. Specifically, the 5 most important features for the final LGA prediction model were paternal ALT ahead of pregnancy, maternal Cr ahead of pregnancy, paternal work/life pressure ahead of pregnancy, paternal heartrate ahead of pregnancy, and paternal Cr ahead of pregnancy.

## Discussion

This study presents a potential clinical tool for a prenatal prediction of LGA births in women exposed to radiation ahead of pregnancy. Six methods were utilized to develop prenatal prediction models with LGA for these women. Compared with conventional LR methods, ML algorithms have better performance in LGA prediction. Thereinto, the RF algorithm developed a more effective prediction model reaching an average AUC value of 0.843 in the test set. The top 10 contributed features were chosen by the RFE method, and the concise prediction model based on the 10 features using the RF algorithm also achieved excellent performance with an average AUC of 0.821. The best that we can tell that we are the first to develop and evaluate ML prediction models for LGA in women who are radiation-exposed ahead of pregnancy. A total of 153 features covering maternal, paternal and environmental factors were included in these prediction models, and thereinto, the paternal and environmental factors were the first time to serve as predictors in ML prediction models for LGA.

Many previous studies have proved the relationship between maternal radiation exposure and fetal birth weight. Maternal radiation exposure ahead of pregnancy, such as diagnostic radiography, radiation therapy and environmental ionizing radiation exposure, may induce significant damage in ovary and uterus, causing an increased risk of fetal abnormal birth weight [20, 21, 51, 52]. However, there are still no prediction models for LGA in women with radiation exposure ahead of pregnancy. In this study, we innovatively applied LR approach and five ML algorithms to prenatal prediction models for LGA in that women group. Among these models, the model based on RF methods displayed the most excellent performance in LGA prediction, with an average AUC of 0.843 in the test set, and the models using GBDT, XGBoost and CatBoost had comparable average AUC values (0.725∼0.768). While the model based on traditional LR approach had the lowest average AUC of 0.603, which might be owing to its inherent constraints of not incorporating the potential interactions among multiple predictors. The ML algorithms can discover unknown correlations between features and LGA births from multidimensional and multivariate data, which might reveal trends ignored by researchers using traditional methods [51]. Moreover, the LR approach is sensitive to outliers and requires a large dataset to work well. Thus, the small sample size and the imbalanced dataset in this study may affect the performance of the LR approach. Our finding showed that the ML algorithms showed great potential in LGA prediction ahead of pregnancy, better discrimination than the traditional LR method (average AUC: 0.843 versus 0.603). The prediction models on the bias of ML algorithms might be potentially promising methods for LGA birth prediction in women with radiation exposure ahead of pregnancy.

In this study, REF method, a wrapper-based backward elimination technique, was performed to rank the most contributed feature [53]. The top 10 contributed features include maternal risk factors (Cr levels, ALT levels, HBeAg, DBP, meat/eggs diet and thyroid examination) and paternal risk factors (Cr levels, ALT levels, heart rate, work/life pressure). The concise model based on these 10 simple features achieved excellent performance with an average AUC of 0.821. In other words, ML algorithms can predict LGA births well using accessible parental physical examination and clinical test indexes. These features’ impact distribution on the output of the final model was represented as the SHAP values in Fig. 4. For example, parental Cr levels, parental ALT levels, paternal work/life pressure and paternal heart rate had positive effects on the LGA prediction outcome. On the contrary, maternal DBP levels had a negative effect on the birth weight of newborns. Specifically, the ALT levels and Cr levels are two commonly used clinical indicators for hepatic and renal function, and the relationships between hepatic/renal function and birth weight of newborns were reported previously [54–57]. Maternal chronic HBV infection also increased the risk of LGA births [58]. Moreover, maternal meat/egg diet means more protein intake. Many studies reported that maternal high protein diet increased birth weight, independently of maternal age, BMI or energy intake, and 1 g protein intake increases the birth weight of newborns by 7.8–11.4 g [59–61]. Additionally, the negative correlation between maternal DBP levels and birth weight of newborns was also reported previously, which was consistent with our study [62, 63]. And the changes of the above features caused by radiation exposure have been reported previously [64–66].

Some previous studies had established prediction models in general population, using ML algorithms or LR approach [25–28]. These models included maternal risk factors, including maternal demographic characteristics, clinical test indexes and ultrasound biometrics measurements. However, most of them performed in prediction for LGA poorly with an average AUC of 0.6∼0.8. In addition to the known maternal risk factors, it was found that birth weight was also associated with paternal risk factors [32, 67, 68]. This study innovatively included paternal risks factors and environmental factors into the prediction models. The results showed that paternal work/life pressure, heart rate and some clinical test indexes were selected as the top 10 contributed features, which showed the indispensable impact of paternal factors in LGA prediction. Unfortunately, the influence of paternal factors on fetal birth weight had received little attention in the past, which might decrease accuracy and applicability of their models.

The current study has several limitations. The data were selected from the NFPHEP project, which were obtained nationally, representing the population with minimal selection bias. However, the small sample size (*n* = 455) and imbalanced (LGA 9.23% vs. non-LGA 91.77%) dataset potentially introduced some other bias. Firstly, the small sample size and imbalanced dataset would cause a large variation in the 95% CI and the low AUC lower bound, which may influence the stability of ML prediction models. Also, due to the limited number of real positive samples, relatively few of the predicted positive samples were actually positive, resulting in a high error rate and a decrease in the accuracy of the positive predictions, reflected as a low PPV. Besides, Bootstrap and Repeated cross-validation were not used in this study, because the number of positive outcomes in the dataset was too small to meet the statistical requirement. Furthermore, training and testing results showed a discrepancy in performance in these models, which indicated potential overfitting. Increasing the sample size was one of the effective ways to mitigate overfitting. In a word, increasing sample size and more balanced datasets would contribute to the development of more high-quality predictive models.

Additionally, for the women with radiation exposure, small-for‐gestational‐age births are more common than LGA births in those offspring with abnormal birth weight. Both small‐for‐gestational‐age and LGA birth prediction are critical topics in obstetrics. However, no LGA prediction model was established before in women with radiation exposure, that’s why we develop and evaluate ML models for LGA prediction in these women. Moreover, as this is a secondary analysis based on NFPHEP project, there was no opportunity to collect additional characteristics. The type or average daily exposure of maternal radiation exposure before pregnancy and ultrasound biometrics measurements during pregnancy were not collected in the dataset, and the above information might improve the performance of ML prediction models. In future work, additional characteristics such as ultrasound biometrics measurements can be included into the models to improve the models’ accuracy, and more validation and application in real world are still required.

In conclusion, six algorithms were utilized to develop the LGA prediction models in women exposed to radiation ahead of pregnancy. After feature selection and optimization approaches, the RF algorithm model based on the top 10 contributed features achieved an average AUC of 0.821, which demonstrates that ML algorithms have a good performance in LGA prediction using parental physical examination and clinical test indexes. Thus, the prediction model using ML algorithms could be a promising tool for prenatal prediction of LGA births in women with radiation exposure before pregnancy.

### Electronic supplementary material

Below is the link to the electronic supplementary material.

Supplementary Material 1

## Data Availability

Our research data were derived from the National Free Preconception Health Examination Project (NFPHEP). Requests to access these datasets should be directed to Hui Pan, panhui20111111@163.com.

## Abbreviations

AUC: Area Under the Curve
BMI: Body Mass Index
CatBoost: Category Boosting
CI: Confidence Interval
GBDT: Gradient Boosting Decision Tree
HBeAg: Hepatitis B Virus e Antigen
IQR: Interquartile Range
LGA: Large-for‐Gestational‐Age
LGBM: Light Gradient Boosting Machine
LR: Logistic Regression
ML: Machine Learning
NFPHEP: The National Free Preconception Health Examination Project
NPV: Negative Predictive Value
PPV: Positive Predictive Value
RF: Random Forest
RFE: Recursive Feature Elimination
ROC: Receiver Operating Characteristic
SD: Standard Deviation
SHAP: Shapley Additive Explanation
XGBoost: Extreme Gradient Boosting
